# Combination and Selection of Traffic Safety Expert Judgments for the Prevention of Driving Risks

**DOI:** 10.3390/s121114711

**Published:** 2012-11-02

**Authors:** Enrique Cabello, Cristina Conde, Isaac Martín de Diego, Javier M. Moguerza, Andrés Redchuk

**Affiliations:** 1 Department of Computer Architecture, University Rey Juan Carlos, Móstoles 28933, Spain; E-Mails: cristina.conde@urjc.es (C.C.); isaac.martin@urjc.es (I.D.); 2 Department of Statistics and Operations Research, University Rey Juan Carlos, Fuenlabrada 28943, Spain; E-Mails: javier.moguerza@urjc.es (J.M.); andres.redchuk@urjc.es (A.R.)

**Keywords:** driving risks, fusion of judgments, selection of experts, regression

## Abstract

In this paper, we describe a new framework to combine experts' judgments for the prevention of driving risks in a cabin truck. In addition, the methodology shows how to choose among the experts the one whose predictions fit best the environmental conditions. The methodology is applied over data sets obtained from a high immersive cabin truck simulator in natural driving conditions. A nonparametric model, based in Nearest Neighbors combined with Restricted Least Squared methods is developed. Three experts were asked to evaluate the driving risk using a Visual Analog Scale (VAS), in order to measure the driving risk in a truck simulator where the vehicle dynamics factors were stored. Numerical results show that the methodology is suitable for embedding in real time systems.

## 1. Introduction

Driving in an urban environment is always a risky and complex experience [1], more so for a truck driver. It includes dealing with pedestrians, cyclists, delivery trucks, buses, parked cars, one-way streets, *etc*. The presence of these features makes city driving more challenging [2]. Among the problems related with urban traffic we can mention the following: parked cars making streets narrower, suddenly stopping cars, pedestrians or cyclists suddenly entering the truck's path, loading and unloading of passengers from buses, almost perpendicular intersections with constricted space to turn, and stop and go traffic.

The United States Department of Transportation [3] estimates that over 500,000 truck accidents occur every year. Nearly 5,000 people die in truck accidents every year, and in 98% of these accidents, deaths include the driver of the non-truck vehicle. Urban areas are affected by 35.4% of fatal truck accidents where in 14.4% of the cases, the cargo was spilled and in 6.5% there were open flames reported.

Consequently, one key issue is how to estimate the driving risk in such an environment [4]. Accident analysis shows that if the vehicle can initiate an emergency braking 180 milliseconds earlier, this will reduce chances of a hospital stay by 15% [5]. This is a very complex task in which a huge number of factors have to be taken into account. These factors can be split in two parts, one related with the data to be considered and the other is about the definition of risk.

Modern truck vehicles are completely sensorized and could incorporate as many as 70 electronic control units (ECU) for various subsystems [6]. The main one is devoted to the engine control unit (also known as engine control module/ECM); others are used for transmission, airbags, antilock braking/ABS, cruise control, electric power steering/EPS, audio systems, windows, doors, mirror adjustment, battery and recharging systems for hybrid/electric cars, *etc*. Some of these form independent subsystems, but communication among all of them is essential. To communicate all elements the can-bus has been selected by the industry as standard (with small variations within each company). In the literature [7–9], very specific measurements are considered, but the most common are data provided from the bus-can in a real vehicle (for example: speed, forces on the pedals, angle of steering wheel) or a complete set of data in a simulator (for example: distance to the preceding vehicle, lane change time). These data are only a subset of the complex information that a driving simulator can provide. Simulators can provide very rich and complex information about vehicles and their environment. The present paper considers the most common elements transmitted in the bus can with close to real situations developed in a highly realistic simulator.

Therefore, to complete all the elements, we have to consider the definition of driving risk [10]. In our normal life, we have to deal with many risky situations. We learn to drive a vehicle and, at the beginning, a supervisor gives us the initial estimation of risky situations. Driving risk is a complex categorization in which the raw evaluation of data is part of the problem. If we add to this evaluation the background and experience of a traffic safety expert we can obtain a more precise evaluation. Unfortunately in many occasions experts' judgments may be different and may lead to inconsistencies. To overcome this problem, in this work we will explore the fusion of experts' information and the selection of consistent experts which arise as feasible solutions. We understand by fusion of information the combination in a single measure of the information provided independently by each expert involved in a given evaluation process.

The data used in this paper come from the “Intelligent cabin truck for road transport” (CABINTEC) project funded by the Spanish Ministry of Science and Innovation, and the European Union. This project is focused on driving risk reduction for professional drivers (mainly focused on trucks and buses). The three main aspects of traffic safety are considered: road, vehicle and driver. Road and vehicle data are obtained from a highly realistic simulator and the hands of the driver are surveyed to measure their position.

This paper is organized as follows: Section 2 introduces the general framework of the problem at hand. In Section 3, we present the computational framework for the fusion and selection of experts. Section 4 describes the high immersive experimental set-up. In Section 5 the numerical experimentation is carried out and results are shown. Finally, Section 6 presents our conclusions.

## 2. General Framework

Driving is a dynamic activity that involves changes in the values of several variables (speed, brakes, lights, and so on) along time. The values of these variables in a precise instant will determine the actions made by the driver. Formally, let us define an action as the values of a set of variables *X*_1_, …, *X_k_* that are stored from a simulator or real situation at the moment in which the driving conditions are taking place. In the case at hand in this work, the values of these variables are the data collected from the can bus installed in a simulator: the speed of the truck, the revolutions per minute (RPM) of the engine, the angle of the steering wheel (SWA), position of the truck on the road, the slope of the road, the position of the pedals and so on. Thus, an action is a multivariate vector determined by the real values for the whole set of variables measured at the given instant.

Suppose that we have a set of actions (a population) made by a driver. This population includes past actions and it could also include future or potential actions. We assume that the size of the population of actions, *N*, is large. Within a quality framework [11] each action is additionally labeled using a quality level. This is equivalent to the situation where a customer compares his expectations for a certain service with its perceived performance. A good service quality evaluation develops when perceptions exceed or are equal to expectations. Consequently, most approaches try to measure this gap directly. The models explaining quality use the concept of importance [12]. The customer determines all characteristics he expects to receive from the ideal service. Since not all of them are equally important, a weight can be assigned to measure the importance of each characteristic. The quality judgment is constructed by summing up all the characteristics multiplied each by its specific significance (ISO 26362: 2009).

Throughout this work, we will use indistinctly the term “variable”, used mainly in the ITS terminology, and the term “characteristic”, more common in the quality management field. Both terms are equivalent from a statistical point of view.

### 2.1. Methodology for Isolate Experts

In Intelligent Transportation Systems (ITS) we can follow a similar approach by assigning to each action a risk level determined by a traffic safety expert. Let us call *Q_i_* the expert perceived quality of the *i_TH_* action (*i* ranks from 1 to *N*, where *N* is the number of actions). Notice that instead of *Q_i_* we may ask the experts to evaluate the perceived risk *R_i_* of the *i_TH_* action. Thus, *Q_i_* and *R_i_* are closely related: the higher *Q_i_*, the lower *R_i_* will be, that is, high quality actions are intended as low driving risk actions. Now, the relationship between service quality and risk level is clear. High quality driving is equivalent to low risk level.

In the literature, it is common to assume that customer's evaluation will be a function of several characteristics *X*_1_,…,*X_k_* which determine the global evaluation of the service. Let us call *X_i_*_1_,…,*X_ik_* to the evaluations of these characteristics made by the *i_TH_* customer. Then:

(1) $$Q_{i}=f(X_{i1},\ldots ,X_{ik})$$

Notice, that in this approach, the function *f* is common to all the customers. In this traditional model (1), *f* is linearly approximated and therefore:

(2) $$Q_{i}=\sum_{j=1}^{k}w_{j}X_{ij}$$

where the coefficients *w_j_* are weights, so that they must be positive and they must add up to one:

(3) $$\begin{array}{ll} w_{i}\geq 0 & \forall j\\ \sum_{j=1}^{k}w_{j}=1 \end{array}$$

These weights can be considered as measures of the relative importance of characteristic *X_i_* in determining the evaluation of the quality of the service for all the customers, that is, the weights are commons to all customers. The limitations of this model are clear. For instance, different actions should have different weights and the previous model does not take this fact into account (because we only have one common function for the whole model). As a concrete example regarding the ITS problem at hand, consider the characteristic “turn signal”. It is clear that if the truck is stopped (not moving) the importance of this characteristic will be low. However, if the truck is moving along a highway and trying to change lanes, this characteristic will be relevant to the driving risk. Therefore our approach should go a step further, because if the expert considers that this lane change is of low risk (for instance because there are no vehicles around), the weight assigned to this characteristic should be also low.

In order to adapt and extend this quality model to measuring driving risk, from now on, we will refer to customers as traffic safety experts. In this way our effort will be dedicated to present and develop a methodology to calculate the weights assigned to each variable describing an action taking into account the perception of the action risk provided by an expert, considering that different actions may have different weights for the characteristics. The methodology has been developed taking into account that the final implementation will be embedded in a system that should take the data and obtain results in real time conditions.

For the sake of simplicity in the following description of the model we will consider a single expert assigning the risk to each action. Let us call *X_i_*_1_,…,*X_ik_* to the values of the characteristics describing the *i_TH_* action. Then, we define:

(4) $$R_{i}=f_{i}(X_{i1},\ldots ,X_{ik})$$

where *R_i_* is the risk assigned by the expert to the *i_TH_* action. Notice that in this model, *f_i_* is specific for each action, and the linear approximation of *f_i_* is given by:

(5) $$R_{i}=\sum_{j=1}^{k}w_{ij}X_{ij}$$

where the coefficients *w_ij_* are specific and individual weights for each action, so:

(6) $$\begin{array}{ll} w_{ij}\geq 0 & \forall_{i},\forall_{j}\\ \sum_{j=1}^{k}w_{ij}=1 & \forall_{i} \end{array}$$

Regression models following a similar reasoning have been presented in [13]. It is important to remark that, in the classical approach we had *k* common weights, that is, *w*_1_,…,*w_k_*, for the whole set of actions. With our approach we have *Nxk* total weights, that is, *k* weights per action.

This methodology also overcomes the traditional consideration of the expert's evaluation of an action. Within the classical settings the whole set of correlations among the characteristics and the risk values provided by the expert are explicitly calculated and used to detect the relevant variables. Under our approach these correlations are implicitly taken into account when calculating the weights for each action. In this way more information is achieved as in addition to the raw correlations, we obtain the explicit important of each characteristic within each action.

### 2.2. Methodology for the Fusion and Selection of Experts

A motivation to use a fusion of judgments is the minimization of errors due to the experts. It is expected that the inclusion of different criteria (experts) will minimize erroneous appreciations coming from some of the experts. As mentioned above, due to possible inconsistencies among different experts, it is interesting to merge all the information or opinions available into a single one [14]. This is an open field of research in ITS. A very preliminary proposal was included in [15]. However in that work, the importance given to each expert was assigned following a uniform distribution, therefore assigning equal importance to all opinions. As remarked by the authors of [15], it was out of the scope of that work the determination of the importance of the experts. Certainly, experts are different depending on their experience, expertise, consistency, *etc.*, and a natural way to merge the opinions of the experts is to use a weighted sum of their evaluations. Thus, we can define the output variable driving risk, “*RiskLevel*” as:

(7) $$\text{RiskLevel}=\sum_{j=1}^{n}a_{i}{\text{Risk}}_{j}$$

where *n* is the number of experts, *α*_j_ is the weight assigned to expert *j*, and *Risk_j_* is the individual risk level evaluation provided by expert *j*.

The weights *α*_j_ should be high for the most confident experts and small for inconsistent experts. In addition:

(8) $$\begin{array}{cc} \sum_{j=1}^{n}a_{j}=1, & 0\leq a_{j}\leq 1 \end{array}$$

In fact, a possibility is to assign the *α*_j_ values from our knowledge of each expert confidence. If no *a priori* knowledge is available, equal weights can be assigned to each expert, with the only condition that the sum of the weights should be equal to one [15]. In the present work, we will explore how to assign weights to the experts depending on the consistency of their judgments. More details will be given in the following sections.

## 3. Computational Framework

In this section we describe how to compute both, the weights *w_ij_* corresponding to the characteristics involved within an action and the weights *a_i_* assigned to each expert.

### 3.1. Numerical Computation of the Characteristics Importance

For the sake of completeness, we include next a description of the algorithm used to calculate the weights described in the previous section. From a mathematical point of view, our initial hypothesis is that there is a function *f_i_*|*R_i_* = *f_i_*(*X_i_*_1_,…,*X_ik_*) for each *i*.

Thus, our model can be locally linearly approximated as $R_{i}=\sum_{j=1}^{k}w_{ij}x_{ij}$. So, we can define the matrix:

(9) $$W=\left[\begin{array}{c} w_{1}\\ \vdots \\ w_{N} \end{array}\right]=\left[\begin{array}{ccc} w_{11} & \cdots & w_{1k}\\ \vdots & \ddots & \vdots \\ w_{N1} & \cdots & w_{NK} \end{array}\right]$$

where *w*_1_, ,*w_N_*, are the vectors corresponding to the rows of *W*, that is, the vectors containing the weights of the characteristics within each action.

#### Definition 1: *ε* -reasonable neighbors

Given an action *X_i_* = *X_i_*_1_,…,*X_ik_*, we will say that *Y* is an *ε*-reasonable neighbor of *X_i_*, if *Y* ∈ *B*(*X_i_*, *ε*), where *B*(*X_i_*, *ε*) denotes the ball of radius *ε* around *X_i_*. It is important to remark that the concept of neighborhood is given by the similarity between two actions. Similar actions may not be consecutive in time. This is one of the key aspects of the method. We take advantage of the whole set of actions available from the past. Two consecutive actions may be very dissimilar even when temporally are close each other. Notice also that the similarity is measured with regards to the value of the characteristics describing the actions and not necessarily the assigned risk. The concept of neighborhood is general enough so that the user of the method may define its own measure.

#### Definition 2: Matrix of reasonable neighbors

Given an action *X_i_* we will choose *l ε*-reasonable neighbors from the whole set of *N* actions, that is, *X*_(1)_,…,*X*_(_*_l_*_)_, where (1),…,(*l*) is an appropriate rearrangement of *l* indexes in the set {1,…,*N*}. For numerical reasons it is advisable to choose *l* ≥ *k*. Thus, we define the matrices *X^i^* and *R^i^* as:

(10) $$X^{i}=\left[\begin{array}{c} X_{i}\\ X_{\left(1\right)}\\ \vdots \\ X_{\left(l\right)} \end{array}\right]=\left[\begin{array}{cccc} X_{i1} & \cdots & \cdots & X_{ik}\\ X_{(1)1} & \cdots & \cdots & X_{(1)k}\\ \cdots & \cdots & \cdots & \cdots \\ X_{(l)1} & \cdots & \cdots & X_{(l)k} \end{array}\right],R^{i}=\left[\begin{array}{c} R_{i}\\ R_{\left(1\right)}\\ \vdots \\ R_{\left(l\right)} \end{array}\right]$$

Notice that we may build *ε* -reasonable neighbors not in the available set of actions. For instance, given an element *X_i_*, if |*ξ*| < *ε*, the element *X_i_* + *ξε* is an *ε* -reasonable neighbors [16].

Regarding the choice of the number of neighbors *l*, it depends on the number of actions available in the data set. It has been chosen as the maximum value between the value *k* and the truncated integer of the value $N^{\frac{4}{k+4}}$ [17].

Our aim is to estimate each component of the matrix *W* with the matrix:

(11) $$\hat{W}=\left[\begin{array}{c} {\hat{w}}_{1}\\ \vdots \\ {\hat{w}}_{k} \end{array}\right]=\left[\begin{array}{ccc} {\hat{w}}_{11} & \cdots & {\hat{w}}_{1k}\\ \vdots & \ddots & \vdots \\ {\hat{w}}_{N1} & \cdots & {\hat{w}}_{Nk} \end{array}\right]$$

**Figure 1.**
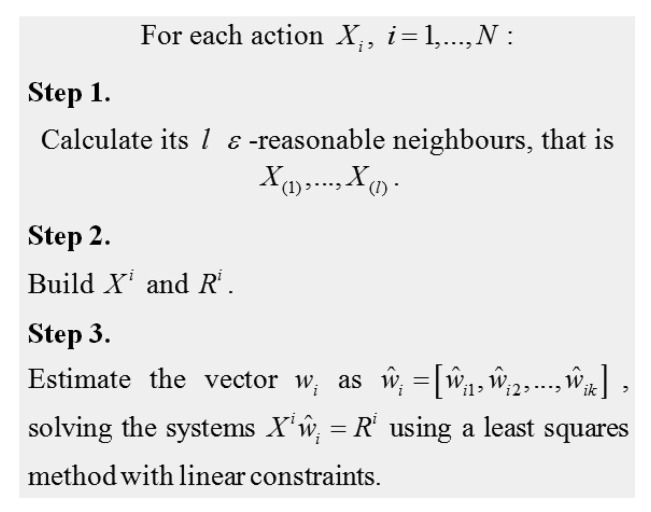
Algorithm to estimate *ŵ_ij_*.

Figure 1 describes in detail how to obtain the matrix *Ŵ*.

We estimate the weights that each action assigns to each characteristic with the information obtained from similar actions. We choose the set of “similarities” based on the nearest neighborhood estimate. This methodology is applied for each expert involved in the evaluation. Once the methodology has been applied, we can obtain an estimation of the expert judgment, *R̂_i_* = *X^i^ŵ_i_* for each action i.

The proposed methodology presents several advantages:

1. When the decision maker needs a single common index, a scalar measure to summarize the performance, we can define:
  (12) $${\hat{w}}_{j}=\frac{\sum_{i=1}^{N}{\hat{w}}_{ij}}{N}$$
2. We have estimated each component *ŵ_ij_*. Based on these estimations, we can use any kind of multivariate method to determine new groups of actions, such us a posterior segmentation, and then infer new results.
3. We define a vector of weights for each action; therefore we are implicitly defining the importance given by the expert to each characteristic of the action. Notice that working with these weights as data we may define new relations among the data.
4. The use of neighbors helps to minimize the simulator errors. In this regard, given that neighbors are chosen using a similarity measure, it is expected that actions including variables with a high acquisition error will be discarded and only actions similar to the current driving situation will be selected. If the acquisition error appears in one variable within the current action, given that the similarity measure takes into account the whole set of variables involved, the use of multivariate actions will minimize its influence.

#### Example 1: A toy example

Let us consider a simple explicative example of how the described methodology calculates the nearest neighbors and the estimation of the judgment for a new action that has not been previously judged. For the sake of simplicity, let us consider that the number of variables describing an action is two, namely, speed and steering wheel angle (SWA). Table 1 shows a sample of five actions with their respective judgments and an unjudged action whose judgment has to be predicted and therefore is not available (NA in the table).

First of all, we have to decide the number of nearest neighbors that are to be calculated. As already mentioned we will use the truncated integer value of $N^{\frac{4}{k+4}}$. In this case the number of actions is *N* = 5 and the number of variables is *k* = 2, leading to the value of $N^{\frac{4}{k+4}}=2.92$. Truncating to the integer we should calculate two neighbors. For the unjudged action we calculate its two nearest neighbors among the five actions using the Euclidean distance. In this case, these two neighbors are Action 3 and Action 5. This corresponds to Step 1 in Figure 1. Notice that to calculate the nearest neighbors only the values of Speed and SWA are needed. Next, using Action 3 and Action 5 and their respective judgments (*R*_3_ and *R*_5_) we can estimate the weights corresponding to the unjudged action, *ŵ_UA_* = [*ŵ_UA_*_1_, *ŵ_UA_*_2_] (Step 3 in Figure 1) by solving the system:

$$\begin{array}{c} 40={\hat{w}}_{UA1}60+{\hat{w}}_{UA2}30\\ 39={\hat{w}}_{UA1}70+{\hat{w}}_{UA2}20 \end{array}$$

These weights are then used to predict the judgment of the unjudged action *R̂_UA_* = 68*ŵ_UA_*_1_ + 25*ŵ_UA_*_2_ =40.7. The same procedure can be used to estimate the predicted risks *R̂_i_*, *i* = 1,…,5 which can be compared with the real risks *R_i_*, *i* = 1,…,5 provided by the experts. These comparisons allow quantifying the prediction errors using actions where real judgments are known.

### 3.2. Numerical Computation of the Experts' Importance

We can define, the mean quadratic error (MQE) for an expert as:

(13) $$\text{MQE}=\frac{\sum_{i=1}^{m}{\left(R_{i}-{\hat{R}}_{i}\right)}^{2}}{m}$$

where *m* is the size of the test sample used. The concept of test sample will be explained in the numerical results section.

Notice that the more consistent the judgments of an expert are, the less value for MQE is. From this definition of MQE, we can build the weights *a_i_* in the following way:

(14) $$\alpha_{i}=\frac{\frac{1}{{\text{MQE}}_{i}}}{\sum_{i=1}^{n}\left(\frac{1}{{\text{MQE}}_{i}}\right)}$$

where *MQE_i_* is the mean quadratic error (MQE) corresponding to expert *i*, *i* = *1*,…,*n*.

It is important to remark that with these definitions the following properties hold:

- The most confident experts, that is, those with the smallest MQE, will have the highest *a_i_* weights.
- The sum of the *a_i_* equals 1, which, as already mentioned, is a desirable property within weighting schemes.

These two properties are exactly the ones specified in Section 2.2.

Using these weights, the fusion of experts is straightforward following the methodology described in Section 2.2. Regarding the selection of experts, the *a_i_* weights can be sorted decreasingly so that the best expert will be that with the highest weight.

## 4. Experimental Set-Up

**Figure 2.**
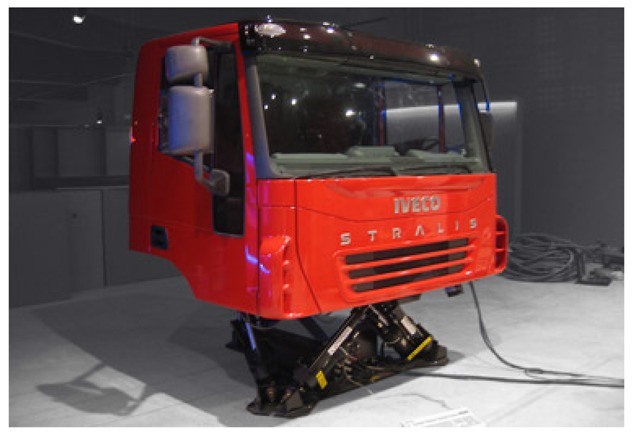
Truck simulator used in the experiments.

**Figure 3.**
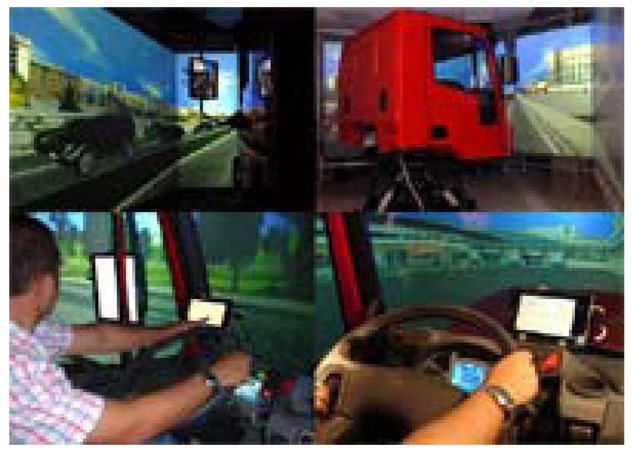
Immersive environment for the simulator.

The methodology was applied to measure the weights of the parameters involved in the driving risk evaluation. As already mentioned, the data used in this paper come from the CABINTEC project. Within this project, a truck cabin simulator has been built. The simulator is a real truck cabin placed on pneumatic actuators and computer controlled, as shown in Figures 2 and 3. The environment is planned to provide all sensory information in a coordinated way to achieve realistic immersion from the beginning. The feeling is like using a real truck and the users perform a naturalistic driving style.

Driving dynamics and kinematics are simulated in order to obtain realistic driving data. To this aim, 12 professional drivers were employed in the project. No previous information was given to the driver, so natural driver behavior was expected. Each driver performs sessions during a minimum of ten minutes. Information on 27 variables was collected at the simulator, among which we can mention the speed of the truck, the revolutions per minute (RPM) of the engine, the angle of the steering wheel (SWA), position of the truck on the road, the slope of the road, the position of the pedals, etc. Each variable provides a data point every 5 milliseconds, so for each session more than 50,000 data points were collected. Also, to provide more information to the experts, two videos per session were collected, the first one with the images projected to the driver and the second in the truck cabin showing the driver's behavior.

Three independent and extern safety experts of the Spanish Royal Automobile Club (“RACE”) evaluated the risk level at each point assigning a value in a Visual Analog Scale (VAS) according to their individual perception. A VAS is a response scale used to measure subjective characteristics or attitudes that cannot be directly measured. When using VAS, experts specify their subjective level of risk by indicating a position along a continuous line whose length goes from zero to 100 [18].

The background of each expert is the following: Expert 1 has high experience in this kind of evaluations and a strong expertise in traffic safety. Experts 2 and 3 are novel to this kind of evaluation, although they also have experience in traffic safety. The scale used adopts values from zero to 100. This method was chosen because it is a simple method that correlates well with other descriptive scales, it has good sensibility and liability, and it can be easily repeated.

**Figure 4.**
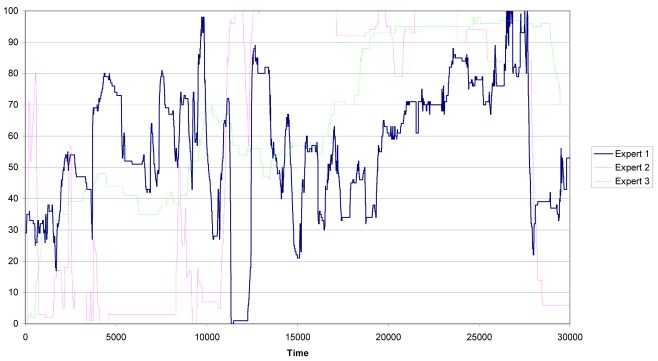
Quantitative evaluation of the risk by the experts.

The experts evaluate the driver's behavior during 10 minutes, which corresponds to 35,000 clock cycles [19]. In the pre-processing of data the first 5,000 clock cycles are eliminated because of instabilities due to warm up and start up of the measurement process. Thus, three evaluations of the risk, one for each expert, from 0 to 100 were acquired. Therefore, each cycle segments an action with the corresponding expert evaluation. The expert evaluations are shown in Figure 4.

It is important to remark that the number of actions amounts to almost 60 per second. This means that we have many similar actions and, if an acquisition error occurs in one action, its influence in the overall performance of the methodology will be very low. Notice also that expert number one (the blue line) presents an evaluation of the risk more stable (with lowest variability) than the evaluation of the risk built by the other two experts (the green and pink lines).

## 5. Numerical Results

In this section we present the results of applying our methodology to the CABINTEC project datasets. We have divided the data corresponding to each expert into two disjoint subsets:

- Training subset: It is used to estimate the weights. The size of this subset is denoted by *n*. So, *n* is the number of adjoin clock cycles selected for training (they are selected starting at the beginning of the session).
- Testing subset: It is used to validate the model established. The size of this subset is denoted by *Tn*. *Tn* is the number of clock cycles used to test.

As described in Section 3, the number of neighbors has been chosen as the maximum value between the value *k* and the truncated integer of the value $n^{\frac{4}{k+4}}$, where *n* is the size of the corresponding training subset and *k* is the number of variables (27 in our case, as described in Section 4). The *ε* -reasonable neighbors have been calculated using the Euclidean distance.

### 5.1. Fusion of Experts

In Table 2 we can see the estimated weight for each expert using different training subset sizes:

It is important to remark that Expert 1 consistently obtains the highest weights. This is coherent with the *a priori* knowledge that we have on the background of each expert (described in Section 4). It is also interesting to remark that as the training size increases, the *a_i_* weights tend to be equal. This is natural since the training error for our methodology is near to zero, and therefore, the larger the training set is, the smaller the error.

**Figure 5.**
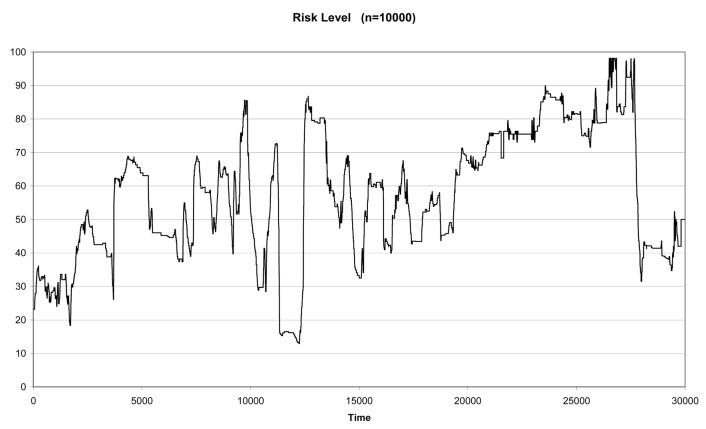
Risk level for the fusion of experts (n = 10,000).

In real situations, our training set will be smaller than the testing set, which corresponds to the infinite possible new actions that may occur in the future. As already mentioned, the methodology has been designed to be embedded within a system that acquires data and make decisions in a real time environment. As a consequence, the most realistic situation is the one that considers a small training set compared to the testing set. This is the case for n = 10,000 and Tn = 20,000. In this situation the *α_i_* weights for experts 1, 2 and 3 are respectively 0.80, 0.10 and 0.10. Figure 5 shows the combined result for these weights.

The methodology described in Section 2 can be used to determine the relevant characteristics for a given expert or fusion of experts. We mean by relevant those characteristics whose *w_ij_* weights in the prediction function have high values. This means that the expert is giving in his evaluation of risk a high importance to the characteristics whose weights are large. Notice that for each characteristic we have a set of *w_ij_* weights, as many as actions are being evaluated. In order to detect which characteristics have the highest weights, we can calculate the median, the quartiles (Q1 and Q3) and the maximum value (Max) for the weights, and then chose those characteristics whose weights correspond to the larger values of these measures (see Table 3). It is important to remark that the first quartile should not be taken as a reference given the large number of actions in which the assigned weight tends to be small. For the fusion of experts with *α_i_* weights 0.80, 0.10 and 0.10, we have calculated the three variables with the highest quartile measures. In order of importance according to the median, these variables are: gear, speed and steering wheel angle.

Notice that the *w_ij_* weights have been assigned using an automatic procedure (the one described in Section 3.1). The only information obtained from the experts is the final risk score assigned to each action. The experts do not provide information about the importance of each characteristic. In our procedure we are able to detect in an automatic way the importance that each expert gives to each characteristic within every single action. This may be seen as a correlation analysis between each variable and the final score given by the experts. The novelty in our methodology is that we calculate explicitly the weight assigned to each variable, and we are able to do this at every instant since the weight changes depending on the action under evaluation.

### 5.2. Selection of Experts

As already mentioned, Expert 1 has the *a_i_* weight with the highest value and therefore it is the expert with the most confident results. Table 4 shows the *a_i_* weights for the three experts for the case n = 10,000.

**Figure 6.**
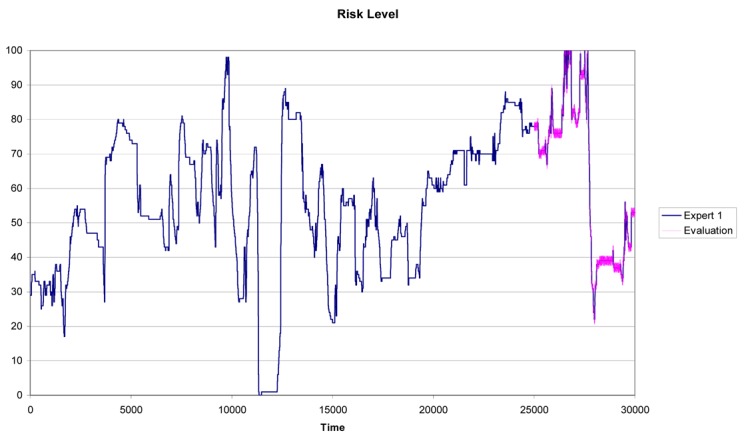
Driving risk evaluation given by Expert 1 (n = 25,000 and Tn = 5,000).

In Figure 6 we can see the results for Expert 1 using a training subset with n = 25,000 and a testing subset with Tn = 5,000. It is clear the similarity between the predicted pattern (the purple line) and the real pattern (the blue line in Figure 6).

Next we have measured the maximum absolute error and the mean square error for different sizes of the training set. Table 5 shows the numerical results. It is apparent that the larger the size of the training set, the smaller the errors are. It is also shown that the maximum absolute error is less that 1.5 (in a scale of 100) even with the smallest training set. It is also important to remark that the variability within the errors is small.

**Figure 7.**
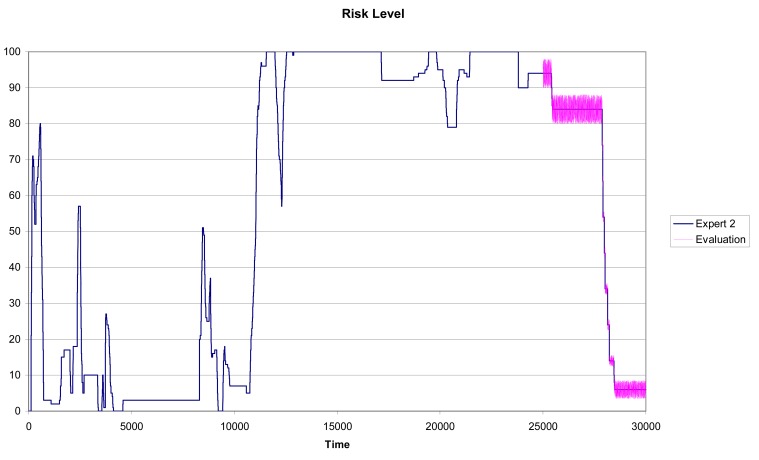
Driving risk evaluation given by Expert 2 (n = 25.000 and Tn = 5.000).

**Figure 8.**
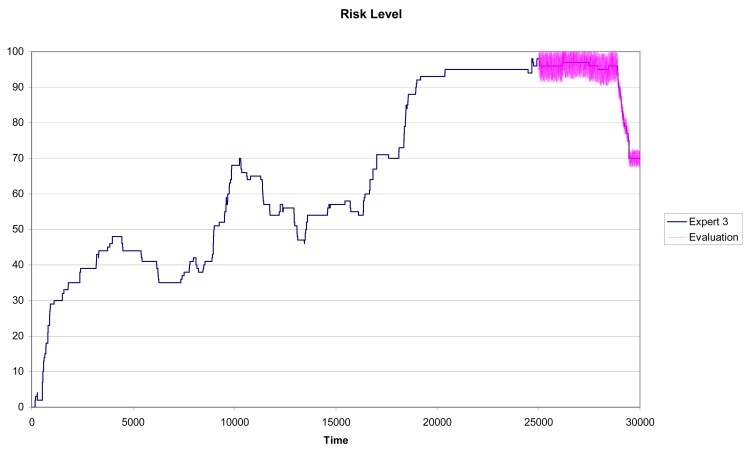
Driving risk evaluation given by Expert 3 (n = 25,000 and Tn = 5,000).

In Figures 7 and 8 we can see the results for Experts 2 and 3 using a training subset with n = 25,000 and a testing subset with Tn = 5,000. In this case, although the similarity between the predicted pattern (the purple line) and the real pattern (the pink line) is high, the prediction results are not as accurate as for Expert 1.

Again for Experts 2 and 3 we have measured the maximum absolute error and the mean square error for different sizes of the training set. Numerical results are shown in Tables 6 and 7. In both cases the errors and the standard deviations are larger than those obtained for the selected expert; that is; Expert 1.

The methodology captures accurately the scoring pattern followed by Expert 1, while Experts 2 and 3 show larger variability in their perceptions of driving risk. Notice that this is confirmed in Figures 7 and 8 as the width of the prediction line (the purple one) is large in some zones of the graph. As described in Section 5.1, for each expert we can calculate the variables with the highest quartile measures. Tables 8, 9 and 10 show the relevant characteristics for each expert.

For Expert 1, the characteristics with the highest values for the median and the third quartile (Q3) are Gear, Steering Wheel angle and Speed. Regarding speed, it is a choice of this expert to assign a high level of risk to this characteristic. This level does not depend on the legal speed limit, but on the adequate speed level according to the driving situation and the expert's judge. Notice that Expert 1 has the most similar results to the ones obtained from the fusion of experts. This is natural since the *α_i_* weight assigned to him has the highest value in the fusion formula.

Regarding Expert 2, the characteristics with the highest values for the median and the third quartile (Q3) are Gear, Lateral position and Speed. Again, the characteristics Gear and Speed appear as some of the important variables. This expert gives also importance to the Lateral position of the truck. The Lateral position is measured with respect to the right lane border.

For Expert 3, the characteristics with the highest values for the median and the third quartile (Q3) are Speed, Lateral position and Accelerator proportion. The Accelerator proportion represents the percentage of acceleration at the given instant (0 means no acceleration and 100 means full gas). As a conclusion, all the three experts agree in the fact that Speed is a relevant characteristic regarding risk.

### 5.3. Applications in the Automobile Industry

The results described in this section follow the current research trends in the automobile industry. The development of safety traffic systems based on real time analysis of variables has been implemented by companies such as Volvo and Mercedes. In this regard Volvo has recently introduced a new technology feature, the so called City Safety. The system has a closing velocity sensor that helps to prevent low speed collisions. [20]. Volvo also uses the system to assist in trailers and towing caravans. In the Volvo case, the main variable analyzed is speed.

Concerning Mercedes, this company has developed the Attention Assist System, which based on the analysis of 70 variables, tends to detect the driver's driving style in order to measure his level of drowsiness [21]. The system was introduced in their 2010 E-class models.

The system developed in this work goes a step further, as it can be used to prevent general risks and is not focused in particular cases (such as drowsiness or low speed collisions). Another novelty is that the system described has been developed using trucks as the basis of the simulation. Traditional safety systems are usually implemented in executive sedan models but it should be taken into account, as already mentioned in the introduction of this paper, the consequences of fatal trucks accidents in urban areas are usually more dangerous than accidents involving sedan models.

## 6. Conclusions

This paper presents a methodology to combine and select experts' judgments for the prevention of driving risks in a cabin truck. The proposed technique allows the calculation of the influence of each characteristic over the final driving risk value, for each expert. This is an important contribution in order to analyze the causes of risky situations during the driving task. In addition, the methodology described in the paper allows the calculation of the explicit weights assigned to characteristics involved in driving risk. Data have been obtained from a truck simulator where the values of the characteristics involved in the vehicle dynamics were stored. Three experts were asked to evaluate the driving risk using a Visual Analog Scale (VAS). We have used the evaluation of each expert to find the weights that he implicitly assigns to every dimension (characteristic) of the given situation (action).

It is important to remark that the methodology emulates the experts' judgments taking into account that the final implementation will be embedded in a system that should take the data and obtain results in real time conditions without the presence of the real experts. Therefore, the methodology has the capacity of being used in a real time scenario.

Particularly, the methodology presented in this work allows the ordering of the experts' judgments according to their relative confidence. Moreover, the characteristics involved in driving risk can also be sorted in order to select the most relevant ones. Notice that *a priori* there are no ground truth variables, that is, the experts have not been asked for the importance that they give *a priori* to each variable. The expert only provides an overall score of risk at each moment. This is the reason why the methodology developed in this work makes sense: the expert does not evaluate each variable; he is quite confident evaluating the whole situation and not isolated variables. Even if this information were available, our methodology will supply us a more accurate value for the weight of each variable within each action. In fact, this knowledge may be used in two ways, a first option is the use of Bayesian methods to include this *a priori* information; a second option is to use this methodology to check if this a priori information is coherent with the real evaluation made by the expert. An interesting further task to explore is the use of methods for time series analysis, given that our experimental data can be seen as a temporal series in which similar sets of actions might reveal something new.

## Figures and Tables

**Table 1.** Five judged actions and a new action whose judgment is not available (NA) in advance and has been predicted.

**Action**	**Speed**	**SWA**	***R****_i_*
Action 1	80	0	21
Action 2	50	90	60
Action 3	60	30	40
Action 4	80	30	54
Action 5	70	20	39
Unjudged Action	68	25	NA

**Table 2.** Estimated weights for each expert using different training sizes, *n* is the number of adjoin clock cycles selected for training.

	**n = 25,000**	**n = 20,000**	**n = 15,000**	**n = 10,000**
Expert 1	0.35	0.37	0.67	0.80
Expert 2	0.33	0.29	0.17	0.10
Expert 3	0.32	0.34	0.16	0.10

**Table 3.** Relevant characteristics for the Fusion of Experts (*n* = 10,000).

	**Standard Deviation**	**Q1**	**Median**	**Q3**	**Max**
Gear	0.11	0.12	0.19	0.27	0.45
Steering wheel angle	0.09	0.08	0.12	0.18	0.48
Lateral position	0.09	0.00	0.08	0.17	0.26
Accelerator prop.	0.12	0.00	0.10	0.22	0.44
Speed	0.12	0.03	0.14	0.23	0.45
Brake prop.	0.06	0.00	0.01	0.06	0.47

**Table 4.** Weights for the experts (n = 10,000).

	*a_i_*
Expert 1	0.80
Expert 2	0.10
Expert 3	0.10

**Table 5.** Results for Expert 1 with n = 25,000; n = 20,000; n = 15,000 and n = 10,000.

	**n = 25,000**	**n = 20,000**	**n = 15,000**	**n = 10,000**
max (abs. error)	1.05	1.28	1.42	1.46
standard dev.	0.35	0.44	0.47	0.51
mean sq. error	0.36	0.55	0.68	0.73

**Table 6.** Results for Expert 2 with n = 25,000; n = 20,000; n = 15,000 and n = 10,000.

	**n = 25,000**	**n = 20,000**	**n = 15,000**	**n = 10,000**
max (absolute error)	1.26	2.14	4.11	5.63
standard dev.	0.42	0.72	1.40	1.93
mean squared error	0.36	0.71	2.72	5.85

**Table 7.** Results for Expert 3 with n = 25,000; n = 20,000; n = 15,000 and n = 10,000.

	**n = 25,000**	**n = 20,000**	**n = 15,000**	**n = 10,000**
max (absolute error)	1.64	2.43	3.62	5.14
standard dev.	0.56	0.82	1.34	1.68
mean squared error	0.38	0.59	2.91	5.91

**Table 8.** Relevant characteristics for Expert 1.

	**Standard Deviation**	**Q1**	**Median**	**Q3**	**Max**
Gear	0.12	0.10	0.19	0.30	0.49
Steering wheel angle	0.10	0.09	0.14	0.21	0.56
Lateral position	0.08	0.00	0.07	0.15	0.11
Accelerator prop.	0.13	0.00	0.11	0.25	0.51
Speed	0.11	0.03	0.14	0.24	0.43
Brake prop.	0.05	0.00	0.01	0.05	0.40

**Table 9.** Relevant characteristics for Expert 2.

	**Standard Deviation**	**Q1**	**Median**	**Q3**	**Max**
Gear	0.08	0.06	0.13	0.19	0.36
Steering wheel angle	0.02	0.01	0.02	0.04	0.17
Lateral position	0.14	0.00	0.12	0.26	0.54
Accelerator prop.	0.02	0.00	0.01	0.04	0.13
Speed	0.10	0.02	0.12	0.20	0.46
Brake prop.	0.08	0.00	0.01	0.08	0.64

**Table 10.** Relevant characteristics for Expert 3.

	**Standard Deviation**	**Q1**	**Median**	**Q3**	**Max**
Gear	0.02	0.01	0.03	0.05	0.11
Steering wheel angle	0.04	0.04	0.06	0.09	0.30
Lateral position	0.09	0.00	0.08	0.17	0.36
Accelerator prop.	0.08	0.00	0.06	0.14	0.37
Speed	0.10	0.02	0.12	0.20	0.45
Brake prop.	0.05	0.00	0.01	0.04	0.40
